# [3-Bromo­meth­yl-1-(4-methyl­phenyl­sulfon­yl)azetidin-3-yl]methanol

**DOI:** 10.1107/S1600536811048392

**Published:** 2011-11-19

**Authors:** Xiao-Qiang Guo, Hu Zheng, Qing-Rong Qi

**Affiliations:** a Department of Medicinal Chemistry, West China School of Pharmacy, Sichuan University, Chengdu 610041, People’s Republic of China

## Abstract

The asymmetric unit of the title compound, C_12_H_16_BrNO_3_S, contains two independent mol­ecules. In each mol­ecule, the azetidine four-membered ring adopts a nearly planar conformation, the maximum deviations being 0.087 (3) and 0.079 (3) Å. The mean azetidine plane is twisted by 75.2 (2) and 73.6 (2)° with respect to the plane of the benzene ring in the two independent mol­ecules. The crystal packing is stabilized by O—H⋯O hydrogen bonds.

## Related literature

For biochemical properties of related compounds, see: Wuitschik *et al.* (2006 ▶). For background to the title compound and related structures, see: Wuitschik *et al.* (2008 ▶).
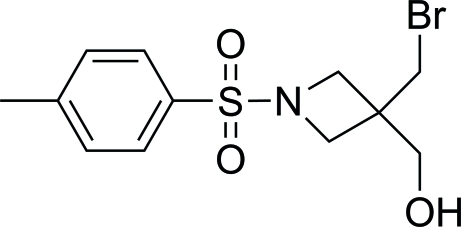


## Experimental

### 

#### Crystal data

C_12_H_16_BrNO_3_S
*M* *_r_* = 334.23
Triclinic, 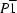

*a* = 6.6290 (9) Å
*b* = 12.4888 (17) Å
*c* = 18.166 (2) Å
α = 109.922 (12)°
β = 95.811 (12)°
γ = 90.199 (12)°
*V* = 1405.5 (3) Å^3^
*Z* = 4
Mo *K*α radiation
μ = 3.07 mm^−1^
*T* = 293 K
0.35 × 0.30 × 0.30 mm

#### Data collection

Agilent Xcalibur Eos diffractometer
Absorption correction: multi-scan (*CrysAlis PRO*; Agilent, 2011 ▶) *T*_min_ = 0.565, *T*_max_ = 1.000
11524 measured reflections
5752 independent reflections
3142 reflections with *I* > 2σ(*I*)
*R*_int_ = 0.042

#### Refinement

*R*[*F* ^2^ > 2σ(*F* ^2^)] = 0.043
*wR*(*F* ^2^) = 0.085
*S* = 0.94
5752 reflections
329 parameters
H-atom parameters constrained
Δρ_max_ = 0.57 e Å^−3^
Δρ_min_ = −0.86 e Å^−3^

### 

Data collection: *CrysAlis PRO* (Agilent, 2011 ▶); cell refinement: *CrysAlis PRO*; data reduction: *CrysAlis PRO*; program(s) used to solve structure: *SHELXS97* (Sheldrick, 2008 ▶); program(s) used to refine structure: *SHELXL97* (Sheldrick, 2008 ▶); molecular graphics: *OLEX2* (Dolomanov *et al.*, 2009 ▶); software used to prepare material for publication: *OLEX2*.

## Supplementary Material

Crystal structure: contains datablock(s) I, global. DOI: 10.1107/S1600536811048392/xu5388sup1.cif

Structure factors: contains datablock(s) I. DOI: 10.1107/S1600536811048392/xu5388Isup2.hkl

Supplementary material file. DOI: 10.1107/S1600536811048392/xu5388Isup3.cml

Additional supplementary materials:  crystallographic information; 3D view; checkCIF report

## Figures and Tables

**Table 1:** Hydrogen-bond geometry (Å, °)

*D*—H⋯*A*	*D*—H	H⋯*A*	*D*⋯*A*	*D*—H⋯*A*
O3—H3⋯O1^i^	0.82	2.01	2.800 (4)	161
O6—H6⋯O4^ii^	0.82	1.94	2.739 (3)	164

Symmetry codes: (i) 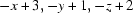; (ii) 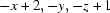.

**Fig. 1.**
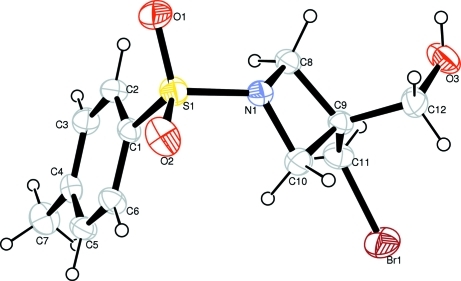
The molecular structure of the title compound with the atom numbering, showing displacement ellipsoids at the 30% probability level.

**Fig. 2.**
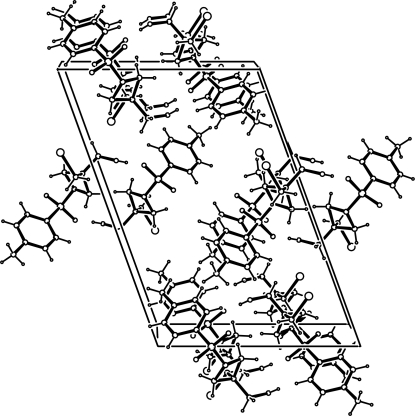
A packing diagram of the title compound.

## supplementary crystallographic information

### Comment

2,6-Diazaspiro [3.3] heptanes may be considered at the very least as a structural surrogate for piperazines. The spirocyclic framework confers upon it the ability to populate structural space not accessible to the parent piperazine. It has potential use as a small-molecule modulator of pharmacokinetic properties (Wuitschik *et al.*, 2006). The title compound, (3-(bromomethyl)-1-(*p*-toluenesulfonyl)azetidin-3-yl)methanol is a important intermediate in our study. So it was synthesized according to the published method (Wuitschik *et al.*, 2008). We report here the crystal structure of the title compound. In the title compound (Fig. 1), the bond angles C10—N1—C8 and C10—C9—C8 are 91.8 (2) ° and 87.2 (3) °, respectively. The crystal packing is stabilized by O—H···O hydrogen bond. The packing view of the title compound is shown in Fig. 2.

### Experimental

The solution of hydrobromic acid (*ca* 33% in AcOH; 6 ml, 35.25 mmol) in Et_2_O (55 ml) was dropwise added to a suspension of 6-(*p*-toluenesulfonyl)-2-oxa-6-azaspiro[3.3] heptane (7.99 g, 31.5 mmol) in Et_2_O (300 ml) at 273 K over a period of 15 min. The resulting mixture was warmed to 298 K and stirred for 45 min. Then the reacting solution was poured into a saturated aqueous solution of NaHCO_3_ (300 ml). The solution were separated and the aqueous phase was extracted with Et_2_O (100 ml). The combined organic layers were dried (MgSO_4_), filtered, and concentrated in vacuo to afford the title compound. Single crystals suitable for X-ray analysis were obtained by slow evaporation of an acetone solution at room temperature.

### Refinement

All H atoms were placed in calculated positions and refined in the riding model, with O—H= 0.8200 Å; C—H= 0.93–0.97 Å. The hydrogen atoms were refined in the riding model with fixed isotropic displacement parameters: *U*_iso_(H) = 1.2*U*_eq_(C) for aromatic, methylene groups, *U*_iso_(H)= 1.5*U*_eq_(C) for methyl group and *U*_iso_(H) = 1.5*U*_eq_(O) for hydroxy groups.

### Crystal data

**Table:**

C_12_H_16_BrNO_3_S	*Z* = 4
*M**_r_* = 334.23	*F*(000) = 680
Triclinic, *P*1	*D*_x_ = 1.579 Mg m^−^^3^
*a* = 6.6290 (9) Å	Mo *K*α radiation, λ = 0.7107 Å
*b* = 12.4888 (17) Å	Cell parameters from 2835 reflections
*c* = 18.166 (2) Å	θ = 2.9–29.1°
α = 109.922 (12)°	µ = 3.07 mm^−^^1^
β = 95.811 (12)°	*T* = 293 K
γ = 90.199 (12)°	Block, colourless
*V* = 1405.5 (3) Å^3^	0.35 × 0.30 × 0.30 mm

### Data collection

**Table:**

Agilent Xcalibur Eos diffractometer	5752 independent reflections
Radiation source: Enhance (Mo) X-ray Source	3142 reflections with *I* > 2σ(*I*)
graphite	*R*_int_ = 0.042
Detector resolution: 16.0874 pixels mm^-1^	θ_max_ = 26.4°, θ_min_ = 3.1°
ω scans	*h* = −8→8
Absorption correction: multi-scan (*CrysAlis PRO*; Agilent, 2011)	*k* = −15→15
*T*_min_ = 0.565, *T*_max_ = 1.000	*l* = −22→22
11524 measured reflections	

### Refinement

**Table:**

Refinement on *F*^2^	Primary atom site location: structure-invariant direct methods
Least-squares matrix: full	Secondary atom site location: difference Fourier map
*R*[*F*^2^ > 2σ(*F*^2^)] = 0.043	Hydrogen site location: inferred from neighbouring sites
*wR*(*F*^2^) = 0.085	H-atom parameters constrained
*S* = 0.94	*w* = 1/[σ^2^(*F*_o_^2^) + (0.014*P*)^2^] where *P* = (*F*_o_^2^ + 2*F*_c_^2^)/3
5752 reflections	(Δ/σ)_max_ = 0.001
329 parameters	Δρ_max_ = 0.57 e Å^−^^3^
0 restraints	Δρ_min_ = −0.86 e Å^−^^3^

### Special details

**Table:**

Geometry. All e.s.d.'s (except the e.s.d. in the dihedral angle between two l.s. planes) are estimated using the full covariance matrix. The cell e.s.d.'s are taken into account individually in the estimation of e.s.d.'s in distances, angles and torsion angles; correlations between e.s.d.'s in cell parameters are only used when they are defined by crystal symmetry. An approximate (isotropic) treatment of cell e.s.d.'s is used for estimating e.s.d.'s involving l.s. planes.
Refinement. Refinement of *F*^2^ against ALL reflections. The weighted *R*-factor *wR* and goodness of fit *S* are based on *F*^2^, conventional *R*-factors *R* are based on *F*, with *F* set to zero for negative *F*^2^. The threshold expression of *F*^2^ > σ(*F*^2^) is used only for calculating *R*-factors(gt) *etc*. and is not relevant to the choice of reflections for refinement. *R*-factors based on *F*^2^ are statistically about twice as large as those based on *F*, and *R*- factors based on ALL data will be even larger.

### Fractional atomic coordinates and isotropic or equivalent isotropic displacement parameters (Å^2^)

**Table:**

	*x*	*y*	*z*	*U*_iso_*/*U*_eq_	
Br1	0.80877 (7)	0.16709 (4)	0.80446 (3)	0.07787 (18)	
Br2	0.33261 (6)	0.14921 (4)	0.31547 (3)	0.07053 (18)	
S1	1.51454 (14)	0.27546 (9)	1.04138 (7)	0.0468 (3)	
S2	1.02857 (14)	0.28105 (9)	0.55348 (7)	0.0487 (3)	
O1	1.6007 (4)	0.3816 (2)	1.09642 (17)	0.0659 (8)	
O2	1.6411 (3)	0.1825 (2)	1.00815 (17)	0.0616 (8)	
O3	1.0699 (4)	0.4627 (2)	0.8377 (2)	0.0793 (10)	
H3	1.1458	0.5191	0.8594	0.119*	
O4	1.1179 (4)	0.2353 (2)	0.61106 (17)	0.0699 (9)	
O5	1.1512 (4)	0.3410 (2)	0.51847 (17)	0.0607 (8)	
O6	0.5704 (4)	−0.1092 (2)	0.3565 (2)	0.0842 (11)	
H6	0.6459	−0.1549	0.3672	0.126*	
N1	1.4048 (4)	0.3079 (2)	0.96791 (18)	0.0415 (8)	
N2	0.9243 (4)	0.1719 (2)	0.48128 (19)	0.0441 (8)	
C1	1.3256 (5)	0.2284 (3)	1.0856 (2)	0.0371 (9)	
C2	1.2236 (5)	0.3063 (3)	1.1414 (2)	0.0477 (10)	
H2	1.2595	0.3836	1.1580	0.057*	
C3	1.0687 (6)	0.2686 (3)	1.1723 (2)	0.0510 (11)	
H3A	1.0004	0.3215	1.2099	0.061*	
C4	1.0108 (5)	0.1538 (3)	1.1490 (2)	0.0448 (10)	
C5	1.1207 (5)	0.0778 (3)	1.0949 (2)	0.0467 (10)	
H5	1.0887	0.0002	1.0796	0.056*	
C6	1.2753 (5)	0.1135 (3)	1.0629 (2)	0.0446 (10)	
H6A	1.3459	0.0607	1.0262	0.054*	
C7	0.8370 (5)	0.1126 (3)	1.1813 (3)	0.0670 (13)	
H7A	0.7259	0.0855	1.1406	0.101*	
H7B	0.7942	0.1743	1.2244	0.101*	
H7C	0.8803	0.0518	1.1996	0.101*	
C8	1.2449 (5)	0.3929 (3)	0.9770 (2)	0.0448 (10)	
H8A	1.2937	0.4670	0.9784	0.054*	
H8B	1.1667	0.4002	1.0207	0.054*	
C9	1.1372 (5)	0.3180 (3)	0.8959 (2)	0.0398 (9)	
C10	1.2804 (5)	0.2230 (3)	0.9007 (2)	0.0480 (10)	
H10A	1.2162	0.1635	0.9145	0.058*	
H10B	1.3515	0.1909	0.8545	0.058*	
C11	0.9156 (5)	0.2929 (3)	0.8966 (2)	0.0542 (11)	
H11A	0.8403	0.3602	0.8989	0.065*	
H11B	0.8969	0.2748	0.9434	0.065*	
C12	1.1785 (6)	0.3631 (3)	0.8315 (2)	0.0558 (11)	
H12A	1.3227	0.3804	0.8352	0.067*	
H12B	1.1387	0.3053	0.7806	0.067*	
C13	0.8356 (5)	0.3687 (3)	0.5967 (2)	0.0409 (9)	
C14	0.7384 (6)	0.3484 (3)	0.6541 (2)	0.0501 (11)	
H14	0.7797	0.2904	0.6727	0.060*	
C15	0.5788 (6)	0.4144 (3)	0.6842 (2)	0.0538 (11)	
H15	0.5129	0.3998	0.7228	0.065*	
C16	0.5157 (6)	0.5015 (3)	0.6578 (2)	0.0472 (10)	
C17	0.6161 (6)	0.5204 (3)	0.6009 (2)	0.0497 (11)	
H17	0.5756	0.5790	0.5827	0.060*	
C18	0.7749 (5)	0.4559 (3)	0.5695 (2)	0.0462 (10)	
H18	0.8402	0.4706	0.5308	0.055*	
C19	0.3394 (6)	0.5712 (3)	0.6908 (3)	0.0725 (14)	
H19A	0.2738	0.5363	0.7222	0.109*	
H19B	0.2442	0.5748	0.6483	0.109*	
H19C	0.3879	0.6469	0.7229	0.109*	
C20	0.7598 (5)	0.0976 (3)	0.4909 (2)	0.0529 (11)	
H20A	0.6813	0.1354	0.5341	0.064*	
H20B	0.8055	0.0251	0.4934	0.064*	
C21	0.6554 (5)	0.0905 (3)	0.4092 (2)	0.0412 (9)	
C22	0.8041 (5)	0.1863 (3)	0.4122 (2)	0.0473 (10)	
H22A	0.8772	0.1687	0.3661	0.057*	
H22B	0.7436	0.2600	0.4235	0.057*	
C23	0.4346 (5)	0.1207 (3)	0.4107 (2)	0.0589 (12)	
H23A	0.4205	0.1881	0.4561	0.071*	
H23B	0.3549	0.0587	0.4155	0.071*	
C24	0.6879 (6)	−0.0218 (3)	0.3458 (3)	0.0580 (12)	
H24A	0.6479	−0.0167	0.2943	0.070*	
H24B	0.8304	−0.0391	0.3489	0.070*	

### Atomic displacement parameters (Å^2^)

**Table:**

	*U*^11^	*U*^22^	*U*^33^	*U*^12^	*U*^13^	*U*^23^
Br1	0.0660 (3)	0.0807 (4)	0.0774 (4)	−0.0317 (3)	−0.0074 (3)	0.0194 (3)
Br2	0.0586 (3)	0.0701 (3)	0.0829 (4)	0.0068 (2)	−0.0081 (3)	0.0307 (3)
S1	0.0379 (6)	0.0522 (7)	0.0537 (7)	−0.0079 (5)	−0.0009 (5)	0.0243 (6)
S2	0.0382 (6)	0.0528 (7)	0.0549 (8)	0.0090 (5)	0.0007 (5)	0.0193 (6)
O1	0.0612 (18)	0.067 (2)	0.065 (2)	−0.0276 (15)	−0.0115 (16)	0.0234 (18)
O2	0.0411 (15)	0.073 (2)	0.084 (2)	0.0173 (15)	0.0200 (16)	0.0407 (19)
O3	0.077 (2)	0.064 (2)	0.108 (3)	−0.0195 (17)	−0.030 (2)	0.056 (2)
O4	0.0580 (18)	0.085 (2)	0.071 (2)	0.0310 (17)	−0.0054 (16)	0.0349 (19)
O5	0.0474 (16)	0.0585 (18)	0.078 (2)	−0.0022 (14)	0.0193 (16)	0.0215 (18)
O6	0.0589 (19)	0.0461 (19)	0.145 (3)	−0.0002 (16)	−0.012 (2)	0.037 (2)
N1	0.0430 (18)	0.0392 (18)	0.042 (2)	−0.0025 (15)	−0.0003 (16)	0.0147 (16)
N2	0.0447 (19)	0.0446 (19)	0.047 (2)	0.0048 (16)	0.0052 (16)	0.0206 (18)
C1	0.034 (2)	0.038 (2)	0.042 (2)	0.0021 (17)	0.0010 (18)	0.0185 (19)
C2	0.053 (3)	0.037 (2)	0.051 (3)	−0.002 (2)	0.002 (2)	0.013 (2)
C3	0.057 (3)	0.046 (2)	0.046 (3)	0.014 (2)	0.010 (2)	0.010 (2)
C4	0.043 (2)	0.056 (3)	0.046 (3)	0.004 (2)	0.006 (2)	0.029 (2)
C5	0.055 (3)	0.036 (2)	0.051 (3)	−0.002 (2)	0.008 (2)	0.017 (2)
C6	0.052 (2)	0.038 (2)	0.049 (3)	0.0093 (19)	0.017 (2)	0.017 (2)
C7	0.059 (3)	0.078 (3)	0.073 (3)	−0.001 (2)	0.021 (3)	0.035 (3)
C8	0.053 (2)	0.038 (2)	0.045 (3)	−0.0023 (19)	0.009 (2)	0.015 (2)
C9	0.039 (2)	0.038 (2)	0.044 (3)	−0.0041 (18)	0.0022 (19)	0.016 (2)
C10	0.055 (2)	0.045 (2)	0.043 (3)	0.000 (2)	0.008 (2)	0.013 (2)
C11	0.049 (2)	0.050 (2)	0.063 (3)	−0.003 (2)	0.013 (2)	0.017 (2)
C12	0.056 (3)	0.065 (3)	0.051 (3)	−0.012 (2)	0.000 (2)	0.028 (2)
C13	0.041 (2)	0.044 (2)	0.039 (2)	0.0041 (18)	0.0009 (19)	0.016 (2)
C14	0.058 (3)	0.052 (3)	0.048 (3)	0.011 (2)	0.008 (2)	0.027 (2)
C15	0.058 (3)	0.063 (3)	0.043 (3)	0.003 (2)	0.018 (2)	0.017 (2)
C16	0.049 (2)	0.042 (2)	0.043 (3)	0.005 (2)	0.003 (2)	0.006 (2)
C17	0.061 (3)	0.042 (2)	0.050 (3)	0.013 (2)	0.010 (2)	0.019 (2)
C18	0.046 (2)	0.048 (2)	0.051 (3)	0.003 (2)	0.016 (2)	0.022 (2)
C19	0.062 (3)	0.070 (3)	0.079 (4)	0.015 (3)	0.020 (3)	0.013 (3)
C20	0.052 (2)	0.051 (3)	0.066 (3)	0.002 (2)	0.011 (2)	0.030 (2)
C21	0.038 (2)	0.039 (2)	0.051 (3)	0.0027 (18)	0.0089 (19)	0.019 (2)
C22	0.056 (3)	0.048 (2)	0.040 (3)	−0.001 (2)	0.009 (2)	0.017 (2)
C23	0.049 (2)	0.054 (3)	0.083 (3)	0.012 (2)	0.015 (2)	0.032 (3)
C24	0.047 (2)	0.044 (2)	0.079 (3)	0.009 (2)	0.010 (2)	0.015 (3)

### Geometric parameters (Å, °)

**Table:**

Br1—C11	1.931 (4)	C9—C10	1.542 (4)
Br2—C23	1.939 (4)	C9—C11	1.503 (4)
S1—O1	1.435 (3)	C9—C12	1.509 (5)
S1—O2	1.431 (2)	C10—H10A	0.9700
S1—N1	1.628 (3)	C10—H10B	0.9700
S1—C1	1.754 (4)	C11—H11A	0.9700
S2—O4	1.434 (3)	C11—H11B	0.9700
S2—O5	1.432 (3)	C12—H12A	0.9700
S2—N2	1.627 (3)	C12—H12B	0.9700
S2—C13	1.754 (3)	C13—C14	1.372 (5)
O3—H3	0.8200	C13—C18	1.386 (4)
O3—C12	1.416 (4)	C14—H14	0.9300
O6—H6	0.8200	C14—C15	1.383 (4)
O6—C24	1.416 (4)	C15—H15	0.9300
N1—C8	1.484 (4)	C15—C16	1.380 (5)
N1—C10	1.484 (4)	C16—C17	1.369 (5)
N2—C20	1.491 (4)	C16—C19	1.509 (4)
N2—C22	1.481 (4)	C17—H17	0.9300
C1—C2	1.380 (5)	C17—C18	1.377 (4)
C1—C6	1.380 (4)	C18—H18	0.9300
C2—H2	0.9300	C19—H19A	0.9600
C2—C3	1.373 (5)	C19—H19B	0.9600
C3—H3A	0.9300	C19—H19C	0.9600
C3—C4	1.390 (5)	C20—H20A	0.9700
C4—C5	1.383 (5)	C20—H20B	0.9700
C4—C7	1.508 (5)	C20—C21	1.546 (5)
C5—H5	0.9300	C21—C22	1.531 (5)
C5—C6	1.371 (5)	C21—C23	1.514 (4)
C6—H6A	0.9300	C21—C24	1.513 (5)
C7—H7A	0.9600	C22—H22A	0.9700
C7—H7B	0.9600	C22—H22B	0.9700
C7—H7C	0.9600	C23—H23A	0.9700
C8—H8A	0.9700	C23—H23B	0.9700
C8—H8B	0.9700	C24—H24A	0.9700
C8—C9	1.549 (5)	C24—H24B	0.9700
			
O1—S1—N1	104.75 (16)	C9—C11—Br1	111.9 (3)
O1—S1—C1	107.73 (18)	C9—C11—H11A	109.2
O2—S1—O1	120.65 (17)	C9—C11—H11B	109.2
O2—S1—N1	106.34 (16)	H11A—C11—H11B	107.9
O2—S1—C1	108.70 (16)	O3—C12—C9	110.7 (3)
N1—S1—C1	108.07 (16)	O3—C12—H12A	109.5
O4—S2—N2	105.38 (16)	O3—C12—H12B	109.5
O4—S2—C13	106.54 (17)	C9—C12—H12A	109.5
O5—S2—O4	120.77 (17)	C9—C12—H12B	109.5
O5—S2—N2	105.93 (17)	H12A—C12—H12B	108.1
O5—S2—C13	109.28 (17)	C14—C13—S2	120.8 (3)
N2—S2—C13	108.40 (16)	C14—C13—C18	119.9 (3)
C12—O3—H3	109.5	C18—C13—S2	119.2 (3)
C24—O6—H6	109.5	C13—C14—H14	120.0
C8—N1—S1	123.0 (2)	C13—C14—C15	119.9 (3)
C10—N1—S1	122.1 (2)	C15—C14—H14	120.0
C10—N1—C8	91.8 (2)	C14—C15—H15	119.5
C20—N2—S2	122.8 (3)	C16—C15—C14	121.1 (4)
C22—N2—S2	121.3 (2)	C16—C15—H15	119.5
C22—N2—C20	91.3 (3)	C15—C16—C19	120.1 (4)
C2—C1—S1	120.1 (3)	C17—C16—C15	117.8 (3)
C2—C1—C6	119.9 (4)	C17—C16—C19	122.1 (4)
C6—C1—S1	120.0 (3)	C16—C17—H17	118.8
C1—C2—H2	120.3	C16—C17—C18	122.5 (3)
C3—C2—C1	119.4 (4)	C18—C17—H17	118.8
C3—C2—H2	120.3	C13—C18—H18	120.6
C2—C3—H3A	119.0	C17—C18—C13	118.8 (4)
C2—C3—C4	121.9 (4)	C17—C18—H18	120.6
C4—C3—H3A	119.0	C16—C19—H19A	109.5
C3—C4—C7	122.0 (4)	C16—C19—H19B	109.5
C5—C4—C3	117.1 (4)	C16—C19—H19C	109.5
C5—C4—C7	120.9 (4)	H19A—C19—H19B	109.5
C4—C5—H5	119.0	H19A—C19—H19C	109.5
C6—C5—C4	122.0 (4)	H19B—C19—H19C	109.5
C6—C5—H5	119.0	N2—C20—H20A	113.8
C1—C6—H6A	120.2	N2—C20—H20B	113.8
C5—C6—C1	119.7 (4)	N2—C20—C21	89.0 (3)
C5—C6—H6A	120.2	H20A—C20—H20B	111.0
C4—C7—H7A	109.5	C21—C20—H20A	113.8
C4—C7—H7B	109.5	C21—C20—H20B	113.8
C4—C7—H7C	109.5	C22—C21—C20	87.4 (3)
H7A—C7—H7B	109.5	C23—C21—C20	113.0 (3)
H7A—C7—H7C	109.5	C23—C21—C22	115.5 (3)
H7B—C7—H7C	109.5	C24—C21—C20	112.4 (3)
N1—C8—H8A	113.8	C24—C21—C22	113.2 (3)
N1—C8—H8B	113.8	C24—C21—C23	113.0 (3)
N1—C8—C9	89.0 (3)	N2—C22—C21	89.9 (3)
H8A—C8—H8B	111.0	N2—C22—H22A	113.7
C9—C8—H8A	113.8	N2—C22—H22B	113.7
C9—C8—H8B	113.8	C21—C22—H22A	113.7
C10—C9—C8	87.2 (3)	C21—C22—H22B	113.7
C11—C9—C8	114.0 (3)	H22A—C22—H22B	110.9
C11—C9—C10	115.5 (3)	Br2—C23—H23A	109.5
C11—C9—C12	113.2 (3)	Br2—C23—H23B	109.5
C12—C9—C8	111.9 (3)	C21—C23—Br2	110.9 (3)
C12—C9—C10	112.5 (3)	C21—C23—H23A	109.5
N1—C10—C9	89.2 (3)	C21—C23—H23B	109.5
N1—C10—H10A	113.8	H23A—C23—H23B	108.1
N1—C10—H10B	113.8	O6—C24—C21	109.3 (3)
C9—C10—H10A	113.8	O6—C24—H24A	109.8
C9—C10—H10B	113.8	O6—C24—H24B	109.8
H10A—C10—H10B	111.0	C21—C24—H24A	109.8
Br1—C11—H11A	109.2	C21—C24—H24B	109.8
Br1—C11—H11B	109.2	H24A—C24—H24B	108.3

### Hydrogen-bond geometry (Å, °)

**Table:**

*D*—H···*A*	*D*—H	H···*A*	*D*···*A*	*D*—H···*A*
O3—H3···O1^i^	0.82	2.01	2.800 (4)	161.
O6—H6···O4^ii^	0.82	1.94	2.739 (3)	164.

Symmetry codes: (i) −*x*+3, −*y*+1, −*z*+2; (ii) −*x*+2, −*y*, −*z*+1.
